# Glycogen Synthase Kinase 3 Protein Kinase Activity Is Frequently Elevated in Human Non-Small Cell Lung Carcinoma and Supports Tumour Cell Proliferation

**DOI:** 10.1371/journal.pone.0114725

**Published:** 2014-12-08

**Authors:** Emma E. Vincent, Douglas J. E. Elder, Linda O′Flaherty, Olivier E. Pardo, Piotr Dzien, Lois Phillips, Carys Morgan, Joya Pawade, Margaret T. May, Muhammad Sohail, Martin R. Hetzel, Michael J. Seckl, Jeremy M. Tavaré

**Affiliations:** 1 School of Biochemistry, Medical Sciences Building, University of Bristol, Bristol, BS8 1TD, United Kingdom; 2 Department of Physiology, McIntyre Building, McGill University, Montreal, Quebec, H3G 1Y6, Canada; 3 Department of Oncology, Hammersmith Campus, Cyclotron Building, London, W12 0NN, United Kingdom; 4 Department of Respiratory Medicine, Bristol Royal Infirmary, Bristol, BS2 8HW, United Kingdom; 5 Division of Histopathology, Bristol Royal Infirmary, Marlborough Street, Bristol, BS2 8HW, United Kingdom; 6 School of Social and Community Medicine, Canynge Hall, University of Bristol, 39 Whatley Road, Bristol, BS8 2PS, United Kingdom; Sun Yat-sen University Medical School, China

## Abstract

**Background:**

Glycogen synthase kinase 3 (GSK3) is a central regulator of cellular metabolism, development and growth. GSK3 activity was thought to oppose tumourigenesis, yet recent studies indicate that it may support tumour growth in some cancer types including in non-small cell lung carcinoma (NSCLC). We examined the undefined role of GSK3 protein kinase activity in tissue from human NSCLC.

**Methods:**

The expression and protein kinase activity of GSK3 was determined in 29 fresh frozen samples of human NSCLC and patient-matched normal lung tissue by quantitative immunoassay and western blotting for the phosphorylation of three distinct GSK3 substrates *in situ* (glycogen synthase, RelA and CRMP-2). The proliferation and sensitivity to the small-molecule GSK3 inhibitor; CHIR99021, of NSCLC cell lines (Hcc193, H1975, PC9 and A549) and non-neoplastic type II pneumocytes was further assessed in adherent culture.

**Results:**

Expression and protein kinase activity of GSK3 was elevated in 41% of human NSCLC samples when compared to patient-matched control tissue. Phosphorylation of GSK3α/β at the inhibitory S21/9 residue was a poor biomarker for activity in tumour samples. The GSK3 inhibitor, CHIR99021 dose-dependently reduced the proliferation of three NSCLC cell lines yet was ineffective against type II pneumocytes.

**Conclusion:**

NSCLC tumours with elevated GSK3 protein kinase activity may have evolved dependence on the kinase for sustained growth. Our results provide further important rationale for exploring the use of GSK3 inhibitors in treating NSCLC.

## Introduction

Lung cancer is the leading cause of cancer death worldwide and non-small cell lung carcinoma (NSCLC) accounts for 85–90% of all cases. The five year survival rate in Europe is 8% [1] and the median survival after diagnosis is 4–5 months if left untreated [2]. Advances in the management of NSCLC using surgery, radiotherapy and chemotherapy have only modestly improved patient survival. The more recent development of the epidermal growth factor receptor (EGFR) tyrosine kinase inhibitors, gefitinib and erlotinib, have provided substantial benefit in a subgroup of patients carrying activating mutations in the EGFR gene [3]. However, this subgroup represents only 10% of all cases of NSCLC in the western world [4] and so there is a continuing need to explore the molecular basis of NSCLC and identify new drug targets for prevention and therapy.

Glycogen synthase kinase 3 (GSK3) was first identified in 1980 as an enzyme that phosphorylated and inactivated glycogen synthase (GS) [5]. There are two isoforms of GSK3, which are semi-redundant and ubiquitously expressed in tissues. The α-isoform encodes a 51 kDa polypeptide and the β-isoform a 47 kDa polypeptide [6], [7]. GSK3 is an unusual kinase in that it is generally active in resting cells and can be inactivated by serine phosphorylation on S21 in GSK3α and S9 in GSK3β [8]. The predominant kinase responsible for this phosphorylation event is Akt, although these sites can also be phosphorylated by PKA, p90Rsk and S6K1 [9]. Growth factors promote glycogen and protein synthesis via phosphorylation and inhibition of GSK3 and the consequent activation of GS and translational initiation factor eIF2B respectively [10]. GSK3 is also phosphorylated on tyrosine residues (GSK3α Y279 and GSK3β Y216) and this activates the kinase [11].

The PI3K/Akt and the Wnt pathways are frequently activated in cancer and both result in inactivation of GSK3 [12]. Consequently the activity of GSK3 has classically been thought to suppress oncogenesis. Indeed, the kinase has been reported to be frequently inactivated in human tumours [13] including oral [14], liver [15] and lung [16]. In contrast, several studies now support the notion that in certain tumour types GSK3 functions to promote tumourigenesis. For example, pharmacological inhibitors of GSK3 block proliferation of various cancer cell lines, such as pancreatic [17], ovarian, [18], [19] mixed lineage leukemia [20] and glioma [21]. Additionally, increased expression and/or activity of GSK3 has been observed in various human cancers including colorectal cancer [22], osteosarcoma [23], renal cell carcinoma [24] and lung [25]. These studies suggest that GSK3 can exhibit both pro-tumour and anti-tumour activity and that this may depend on the tumour type. In none of these studies, however, was GSK3 protein kinase activity systematically examined, which is an important omission given its complex regulation by multisite phosphorylation.

In this study, we explored the role of GSK3 in NSCLC by examining its expression *and* protein kinase activity in freshly isolated NSCLC tissue compared to patient-matched control tissue, and the sensitivity of NSCLC cell lines to inhibition of GSK3 using a small molecule inhibitor. We show that GSK3 expression and protein kinase activity is elevated in 41% of NSCLC tumours and that GSK3 protein kinase activity supports the proliferation of NSCLC cell lines, but not of immortalised pneumocytes. These data suggest the potential for a subset of patients with NSCLC to benefit from the therapeutic use of GSK3 inhibitors previously developed for other human diseases.

## Materials and Methods

### Patients and tissue samples

Ethics statement: Patients with suspected lung cancer were identified from an elective thoracic surgery list at the Bristol Royal Infirmary, Bristol, UK. The study was approved by the local research ethics committee: UK National South West 4 Research Ethics Committee (REC) Southmead Hospital, Bristol BS10 5NB. REC no: 07/Q2002/6, South West 4 REC. Written consent was obtained from all patients. Collection of samples and details of the cohort are the same as previously described [26]. In brief, three distinct samples of the tumour and adjacent normal tissue from the resection margin were taken and flash frozen in liquid nitrogen as quickly as possible (average time taken to freeze samples after surgical removal was 15.9 minutes; range 8–25 minutes). A pathologist recorded the histology and stage of the tumour, and made an assessment of the percentage of each sample that was tumour. Only those NSCLC tumour samples comprising at least 90% tumour tissue were included in the study. All samples were stored at −80°C until required for analysis.

### Materials

The GSK3 inhibitor CHIR99021 (Stemgent, Cambridge, MA, USA #04-0004) was dissolved in DMSO and diluted in culture medium before use. Rabbit polyclonal pCRMP (Thr514) and pGS (S641) antibodies were from Cell Signaling Technology (Boston, MA). Murine anti-F_1_-ATPase and rabbit polyclonal pNFκB p65 (Ser468) antibodies were from Santa Cruz Biotechnology Inc. (Santa Cruz, CA), murine anti-GSK3α/β antibody was purchased from Millipore (Millipore, Hertfordshire, UK), and donkey horse-radish peroxidase-conjugated anti-mouse IgG and anti-rabbit IgG antibodies were from Jackson ImmunoResearch Laboratories (Bar Harbor, ME). Luminex Multiplex Bead Immunoassay kits (Invitrogen Ltd., Paisley, UK; buffer kit #LHBO002) were used to analyse pGS (S641/645) (LHO0731) and GSK3β (LHO0451) in patient samples and Luminex Bio-Plex Phosphoprotein assay kits (Bio-rad, Hemel Hempsted, UK; buffer kit # 171-304004) were used to analyse pGSK3 (S21/9) (#171V23318).

### Cell culture

Hcc193, H1975, PC9 and A549 cells were from the National Institutes of Health NCI-60 panel. Cell lines are regularly tested (every 6 months) using short tandem repeat (STR) profiling by the cell authentication service offered by LGC (Teddington, UK). Type II pneumocytes were generated from primary cultures and isolated as described [27]. The cell lines were cultured in “growth medium” consisting of RPMI 1640 or DCCM-1 (type II pneumocytes) supplemented with 10% foetal bovine serum (FBS, Invitrogen, Paisley, UK), 20000 U/ml penicillin, 7 mM streptomycin and 200 mM glutamine. All cell lines were grown at 37°C in a humidified atmosphere supplemented with 5% (v/v) CO_2_.

For western blotting experiments cells were seeded in 6 or 12 well experimental plates so that the cultures were 90% confluent after 24 h. Cells were then cultured in the presence or absence of CHIR99021 for 24 h. The final DMSO concentration in all cultures was 0.5% (v/v). Cells were then transferred to ice and protein was extracted as previously described [28].

### Growth assays

For analysis of adherent cell growth, cell lines were trypsinised and gently passed through an 18G needle to form individual single cell suspensions. Cells were seeded (1×10^3^ cells/well) in 96 well plates in RPMI 1640 containing 10% (v/v) FBS, 100 U/ml penicillin, 0.1 mg/ml streptomycin and 2 mM L-glutamine. After 24 h medium was replaced with medium containing CHIR99021 (1, 5 or 10 µM) or DMSO, with cells treated in triplicate. Cells were incubated for 120 h, at which point 10% by volume of Alamar Blue (Serotec, Kidlington, UK) was added to the wells and the cultures incubated for a further 2–5 hours. Metabolically active cells convert Alamar Blue to a fluorescent indicator so that quantification of fluorescence is a measure of the number of living cells. Fluorescence was analysed using a Perkin Elmer Fusion plate reader with 535 nm excitation and 590 nm emission filters.

### Preparation of tissue lysates

Frozen tissue samples were homogenised and extracted as previously described [26]. In brief, a Polytron homogeniser was used to generate tissue lysates in 1% NP40 lysis buffer. Tissue lysates were rotated end-over-end at 4°C for 30 min, and rested on ice for 15 min before centrifugation at 16,000×g for 15 min to remove insoluble material. Protein concentration was determined by BCA assay (Thermo Scientific, IL, USA) and tissue lysates were stored at −80°C.

### Luminex (xMAP) assays

Multiplex Bead Immunoassays (Invitrogen) and Bio-Plex Phosphoprotein assays (Bio-Rad) were carried out in individual plates with their own buffers and standards following the directions of the manufacturer. Lung tissue lysates were diluted in extraction buffer to 1 mg/ml (Invitrogen) or 0.4 mg/ml (Biorad) followed by dilution to 0.2 mg/ml in assay diluent. Aliquots of 50 µl containing 10 µg of protein were combined with coated beads. Incubations and washes were performed using a vacuum manifold in the 96-well filter membrane plates supplied. Plates were analysed with a Luminex 200^™^ instrument. An acquisition gate was set between 8000 and 13500 for the doublet discriminator for a sample volume of 50 µl, with 50 events/region being acquired. Three separate samples of tumour tissue and three separate samples of normal tissue from each individual patient were analysed in duplicate in each Luminex assay. The median fluorescence intensity was determined. Data are expressed as mean ± SEM, and the strength of evidence for the difference in phosphorylation/expression between the normal and tumour samples was determined by a Kruskal-Wallis rank order test (p<0.05).

### Western blotting analysis

NSCLC cell or tissue lysates (15 µg protein) were subjected to SDS-PAGE and western blotting as previously described [26]. Lysates were separated using 4–12% Bis-Tris gradient gels (Invitrogen Ltd., Paisley, UK), proteins were transferred to polyvinylidene difluoride membranes (Millipore, Hertfordshire, UK) and blocked using 5% (w/v) bovine serum albumin for 1 hour. Membranes were washed, incubated with primary antibody (1 µg/ml) overnight before washing and incubating with the appropriate secondary antibodies for 1 hour. Immunoblots were visualised using an Enhanced ChemiLuminescence detection system (ECL; Amersham Biosciences).

## Results

### Analysis of GSK3 expression in human NSCLC

The cohort used in this study comprised 29 patients undergoing thoracic surgery for suspected lung cancer. Following resection from the patient three samples of tumour were snap frozen in liquid nitrogen. Only tumour samples confirmed by analysis of frozen sections to consist of at least 90% tumour tissue were then retained for use in the study. Three samples of normal lung tissue were also taken from the resection margin surrounding the tumour and snap frozen. The use of patient-matched normal tissue allowed us to determine whether protein expression and phosphorylation was increased, decreased or unchanged in the tumour relative to normal lung from that particular individual.

**Figure 1:**
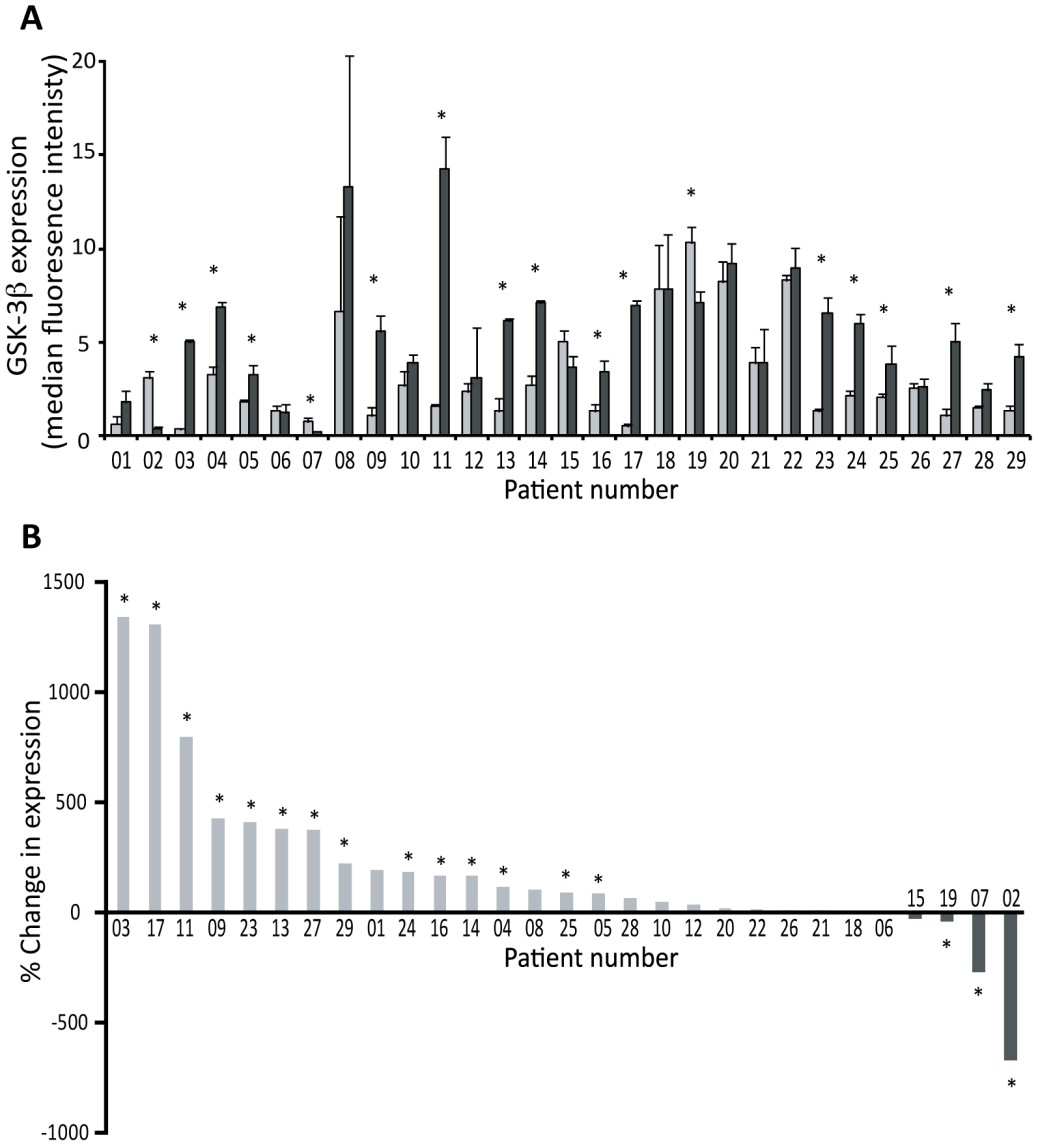
Expression of GSK3 in NSCLC tumour tissue in comparison to patient-matched normal lung tissue. Three distinct samples from patient-matched normal (N1-3) and tumour (T1-3) tissues were analysed using Luminex (xMAP) technology to determine the level of GSK3β expression. A: Quantified data for all 29 patients. Each bar represents the average expression for normal (N1-3; light grey) or tumour (T1-3; black) tissue for each patient (mean ± SEM). The strength of evidence for a difference in expression between the normal and tumour samples was determined by a Kruskal-Wallis test, and * indicates p<0.05. B: The percentage change in GSK3 expression in tumour samples in comparison to patient-matched normal tissue where patients are ranked in order of the extent of the percentage change in expression.

For all 29 patients, each of the three separate samples of tumour and three samples of patient-matched normal tissue were analysed using Luminex (xMAP) technology to assess the level of GSK3 expression and activity in NSCLC tissue in comparison to the normal lung samples. Luminex (xMAP) technology was chosen, as it is a directly quantitative technique in comparison to semi-quantitative western blotting (S1 Figure). Fig. 1A shows data for the expression level of GSK3β in the tumour samples (dark grey bars) compared to samples of patient-matched normal tissue (light grey bars). These data are reorganised in Fig. 1B according to the magnitude of the fold-change; from highest fold-increase to highest fold-decrease in tumour compared to normal tissue.

**Figure 2:**
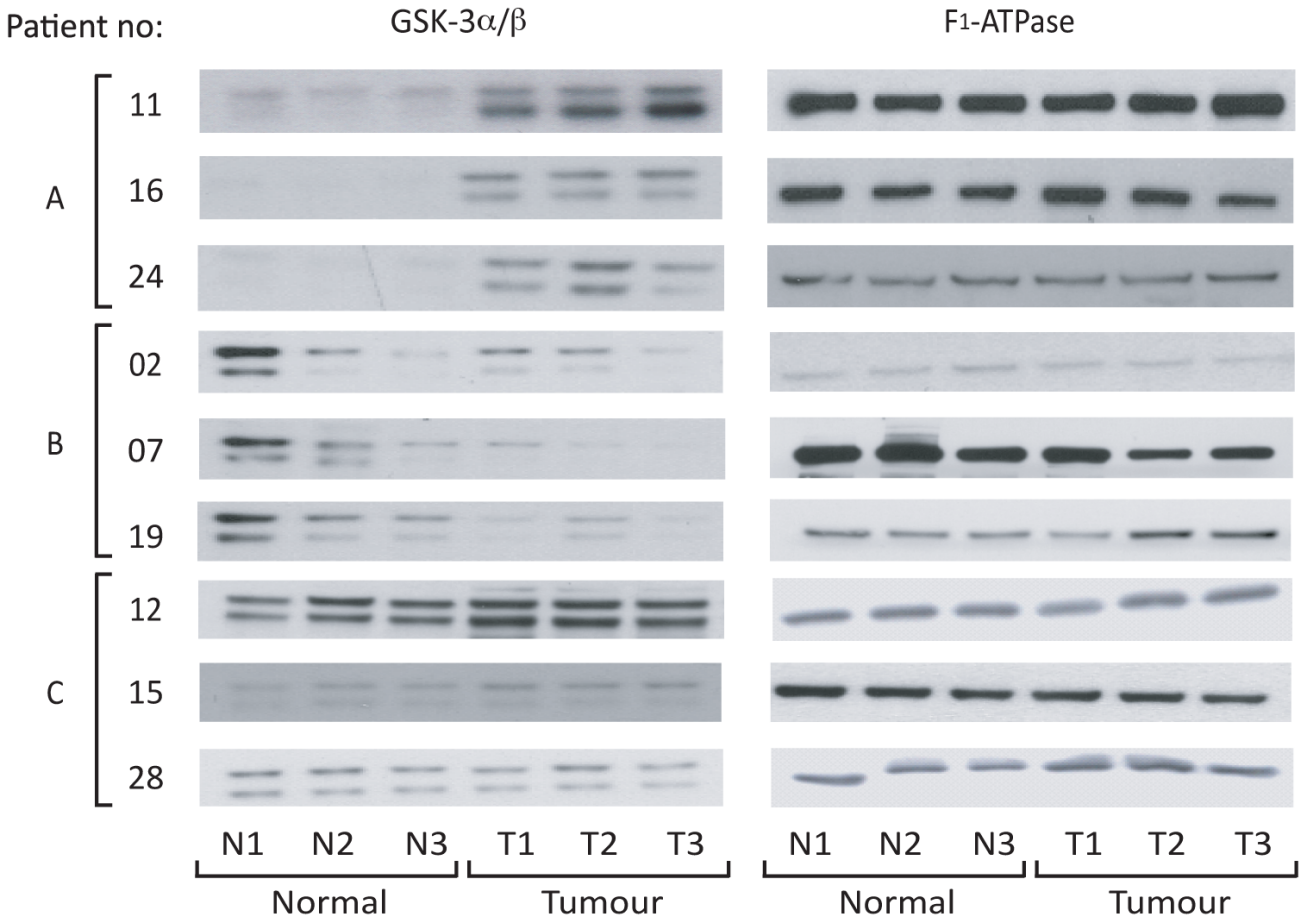
GSK3α/β expression in selected patients. Three distinct samples from normal (N1-3) and patient-matched tumour (T1-3) tissues were separated on SDS-PAGE gels. Expression of GSK3α/β was determined by western blotting. An anti-F_1_-ATPase antibody was used as a control for protein loading.

GSK3β protein expression was increased in 14/29 (48%) of tumours (at p<0.05; Kruskal-Wallis test) when compared to the patient-matched normal tissue, decreased in 3/29 (10%) tumours (p<0.05) and unchanged in the remaining 12 tumours (i.e. p>0.05). Samples from nine patients were further analysed by western blotting with an anti-GSK3 antibody that recognises both the α and β isoforms. Three patients in which GSK3β was increased (Fig. 2A), three where it was decreased (Fig. 2B) and three where it was unchanged (Fig. 2C) were analysed. F_1_-ATPase expression demonstrated equal protein loading. The results support the Luminex data for GSK3β (Fig. 1) and reveal that changes in GSK3β expression were mirrored by equivalent changes in the level of GSK3α.

### Analysis of GSK3 protein kinase activity in human NSCLC

We next assessed whether increased GSK3 expression resulted in increased activity of the protein in NSCLC by analysing GSK3 phosphorylation and downstream signalling. GSK3α/β phosphorylation on S21/9 was analysed in all patients using Luminex technology (S2 Figure). S21/9 phosphorylation, which inhibits the enzyme, was increased in 25/29 (86%) of tumours (at p<0.05; Kruskal-Wallis test) when compared to matched normal tissue and in the remaining 4 tumours was unchanged (i.e. p>0.05). All tumours that exhibited an increase in GSK3 expression by Luminex analysis (Fig. 1) also showed a parallel increase in GSK3 S21/9 phosphorylation. After normalisation of GSK3 S21/9 phosphorylation to the level of GSK3 expression, the net level of serine phosphorylation in all but one of these tumours is no longer increased (S3 Figure). This strongly suggests that the gross increase in S21/9 phosphorylation observed in these tumours is predominantly a reflection of the elevation of GSK3 expression and does not necessarily indicate a decreased activity of the enzyme.

**Figure 3:**
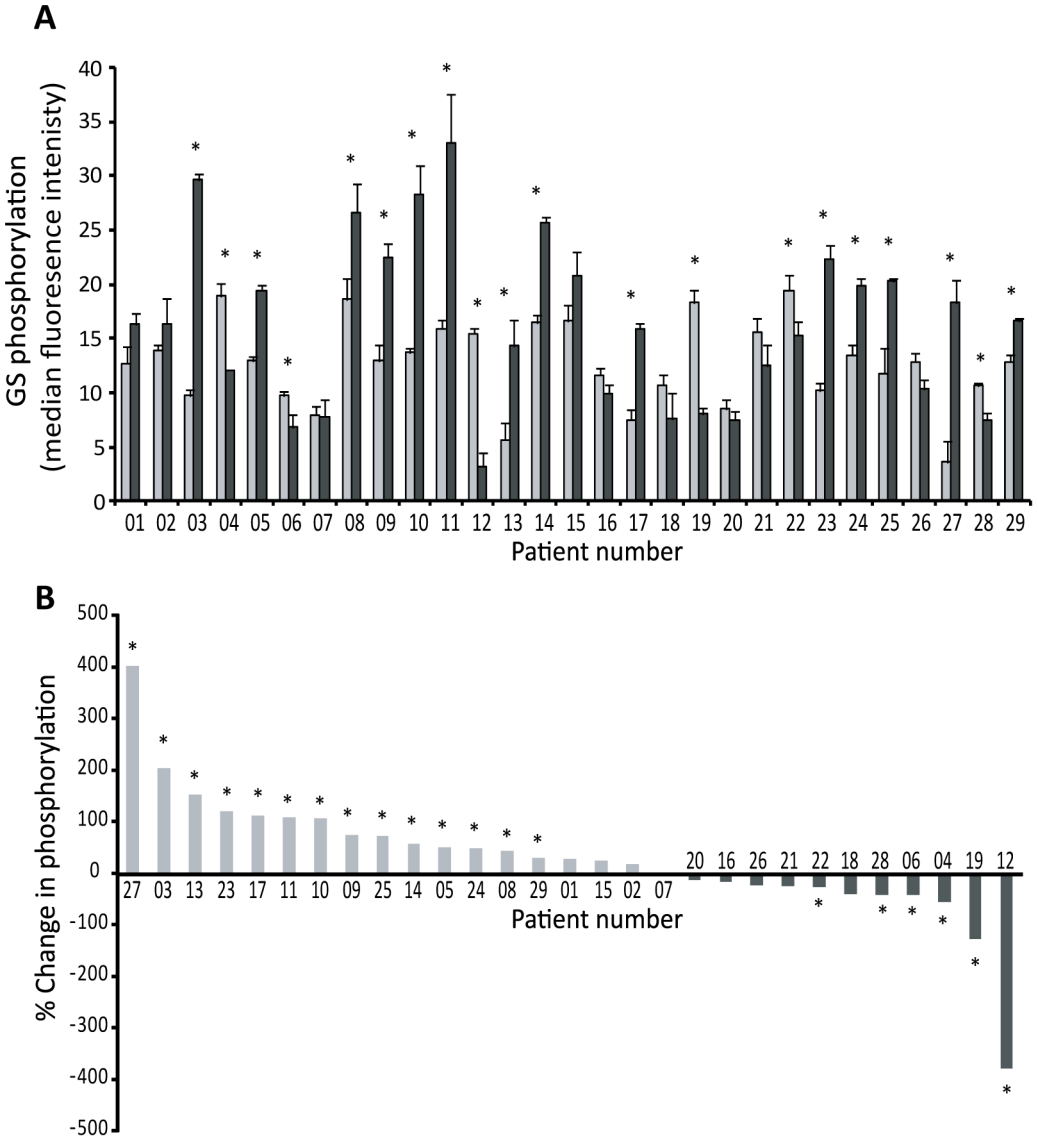
Phosphorylation of GS on S641/645 in NSCLC tumour tissue in comparison to patient-matched normal lung tissue. Samples were analysed using Luminex (xMAP) technology exactly as described in Fig. 1. A: The extent of GS phosphorylation in normal (N1-3; light grey) or tumour (T1-3; black) tissue for each patient (mean ± SEM; * indicates p<0.05). B: Shows the percentage change in GS (S641/645) phosphorylation in tumour samples in comparison to patient-matched normal tissue with patients ranked according to the percentage change in phosphorylation.

To obtain a direct readout of *in vivo* GSK3α/β activity, rather than inferring activity by measuring levels of S21/9, GSK3 activity was determined by measuring the phosphorylation of three distinct and well established downstream GSK3 substrates. The phosphorylation of glycogen synthase (GS) by GSK3 on S641/645 [29] was measured by Luminex detection. Fig. 3A and 3B show that GS S641/645 phosphorylation was increased in 14/29 tumours (48%; p<0.05) when compared to patient-matched normal tissue, decreased in 6/29 (21%; p<0.05) and unchanged (i.e. p>0.05) in the remaining nine tumours.

**Figure 4:**
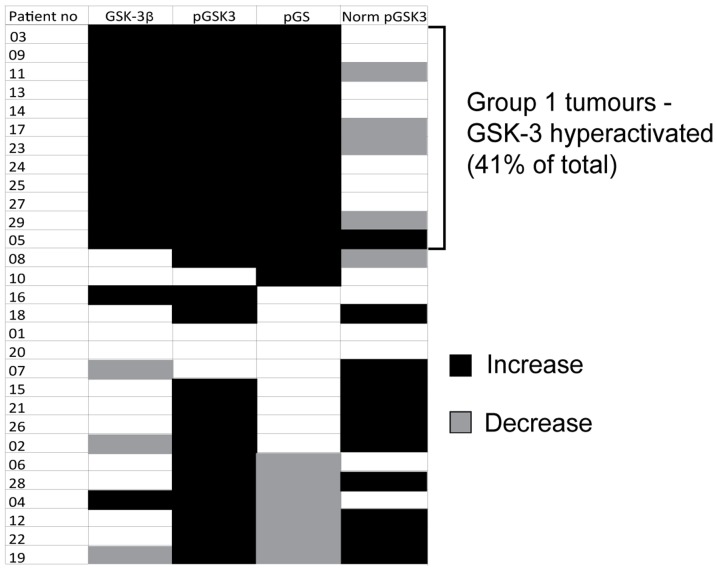
Summary of GSK3 activity in all patients. Three distinct samples from normal and patient-matched tumour tissues were analysed using Luminex (xMAP) technology to determine the level of GSK3β expression, GS phosphorylation (S641/645) and GSK3α/β phosphorylation (S21/9). GSK3 phosphorylation has also been normalised to GSK3 expression. The strength of evidence of difference in expression/phosphorylation between the normal and tumour samples was determined by Kruskal-Wallis test. Expression/phosphorylation of each protein/site was then coded according to whether it increased (black), decreased (grey) at p≤0.05 or remained unchanged (white; p>0.05) in the tumour samples relative to normal tissue. The coded data were then grouped according to the direction of change in GSK3 expression: patients in Group I showed an increase in GSK3 expression and GS phosphorylation.

The data were there then coded for the levels of expression and S21/9 phosphorylation of GSK3, and for GS S641/645 phosphorylation according to whether they increased or decreased at p<0.05. The coded data are represented within the chart shown in Fig. 4 and are grouped according to the direction of change in GSK3β expression and GS phosphorylation. 12/29 (41%) of tumours had a parallel increase in GSK3β expression and GS phosphorylation on S641/645 relative to normal tissue (these tumours are termed ‘Group 1’ in Fig. 4). These data suggest that GSK3 protein activity is elevated in these tumours, despite the gross in S21/9 phosphorylation. Indeed, upon normalisation of GSK3 phosphorylation to total GSK3 the net level of serine phosphorylation is no longer increased in all but one Group 1 tumour.

**Figure 5:**
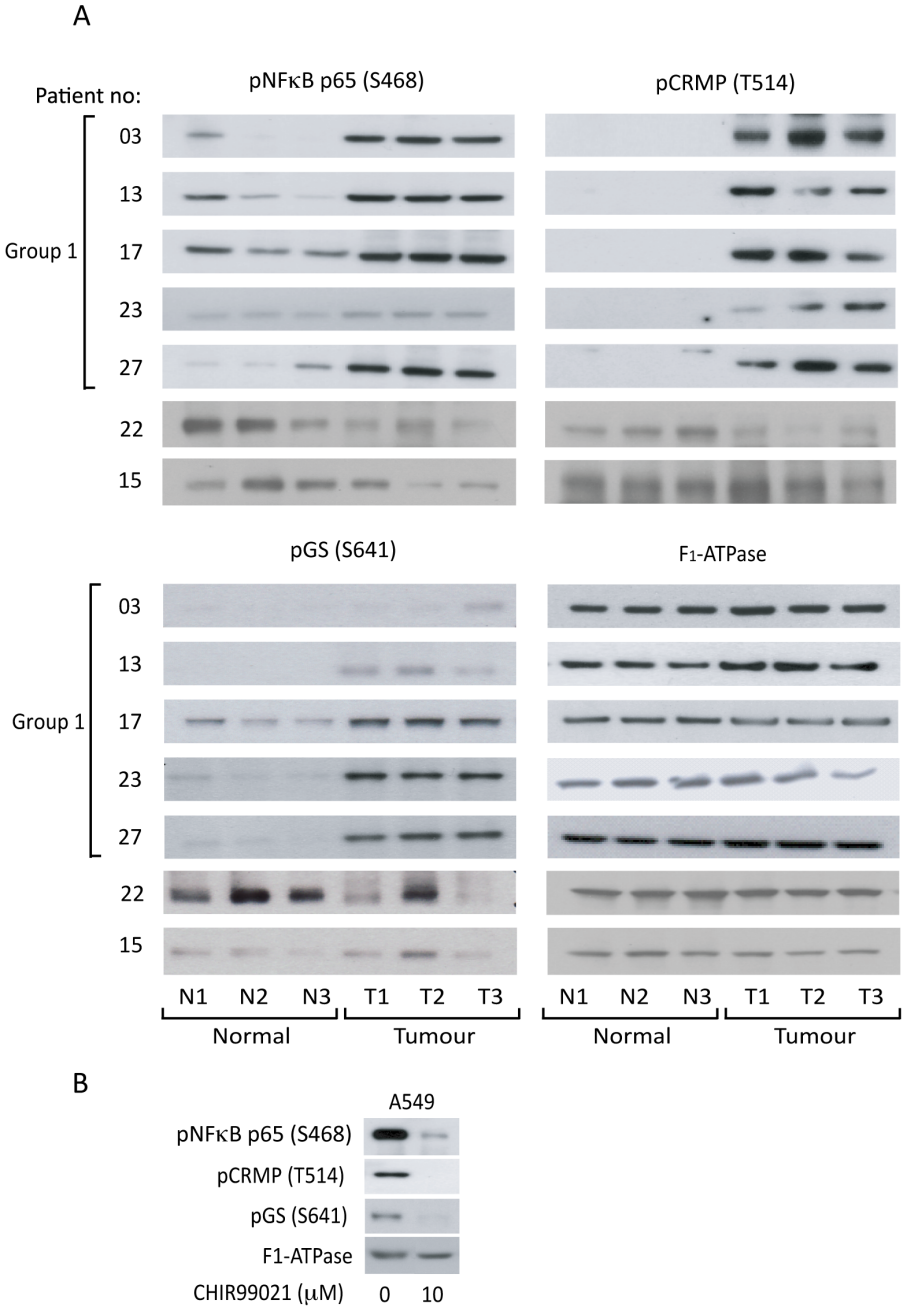
Phosphorylation of downstream GSK3 substrates in selected patients. A: Three distinct samples from normal (N1-3) and patient-matched tumour (T1-3) tissues were separated on SDS-PAGE gels. Expression of GSK3 and phosphorylation of GS (S641), NFκB p65 (S468) and CRMP (T514) was determined by western blotting with specific antibodies as indicated. An anti-F_1_-ATPase antibody was used as a control for protein loading. B: A549 cells were cultured in the absence and presence of 10 µM CHIR99021. Protein was extracted and lysates were separated on SDS-PAGE gels. Expression of GSK3 and phosphorylation of GS (S641), NFκB p65 (S468) and CRMP (T514) was determined by western blotting.

To further investigate the changes in GSK3 protein kinase activity, western blotting was used to analyse a subset of Group 1 tumours (03, 13, 17, 23 and 27) (Fig. 5A) for the phosphorylation of GS and two additional established GSK3 substrates: the p65-RelA subunit of NFκB on S468 [30] and CRMP on T514 [31]. Phosphorylation of all three GSK3 substrates was increased in Group 1 tumour tissue in comparison to normal, and this was particularly marked in the case of CRMP T514 phosphorylation (Fig. 5A). To demonstrate that substrate phosphorylation correlates with GSK3 expression levels we have also included two non-Group 1 tissue samples (patients 22 and 15 have decreased and unchanged expression of GSK3 in their tumour tissue respectively). That these proteins are GSK3 substrates in NSCLC cells was confirmed in the A549 line in which GSK3α/β inhibitor CHIR99021 decreased the phosphorylation of all three substrates (Fig. 5B).

### GSK3 protein activity is required for growth of NSCLC cell lines

As an increase in GSK3 activity in 41% of the NSCLC patient tumours was observed, we explored whether GSK3 protein activity may contribute to tumour cell proliferation in a subset of NSCLC cell lines. We employed a cell-permeant ATP-competitive inhibitor of GSK3; CHIR99021, four NSCLC cell lines (Hcc193, H1975, PC9 and A549) and immortalised normal human lung alveolar cells (type II pneumocytes). CHIR99021 is the most selective GSK3 inhibitor currently available and inhibits both GSK3 isoforms with 350-fold greater potency than it does other cyclin-dependent kinases (CDKs) [32], [33].

**Figure 6:**
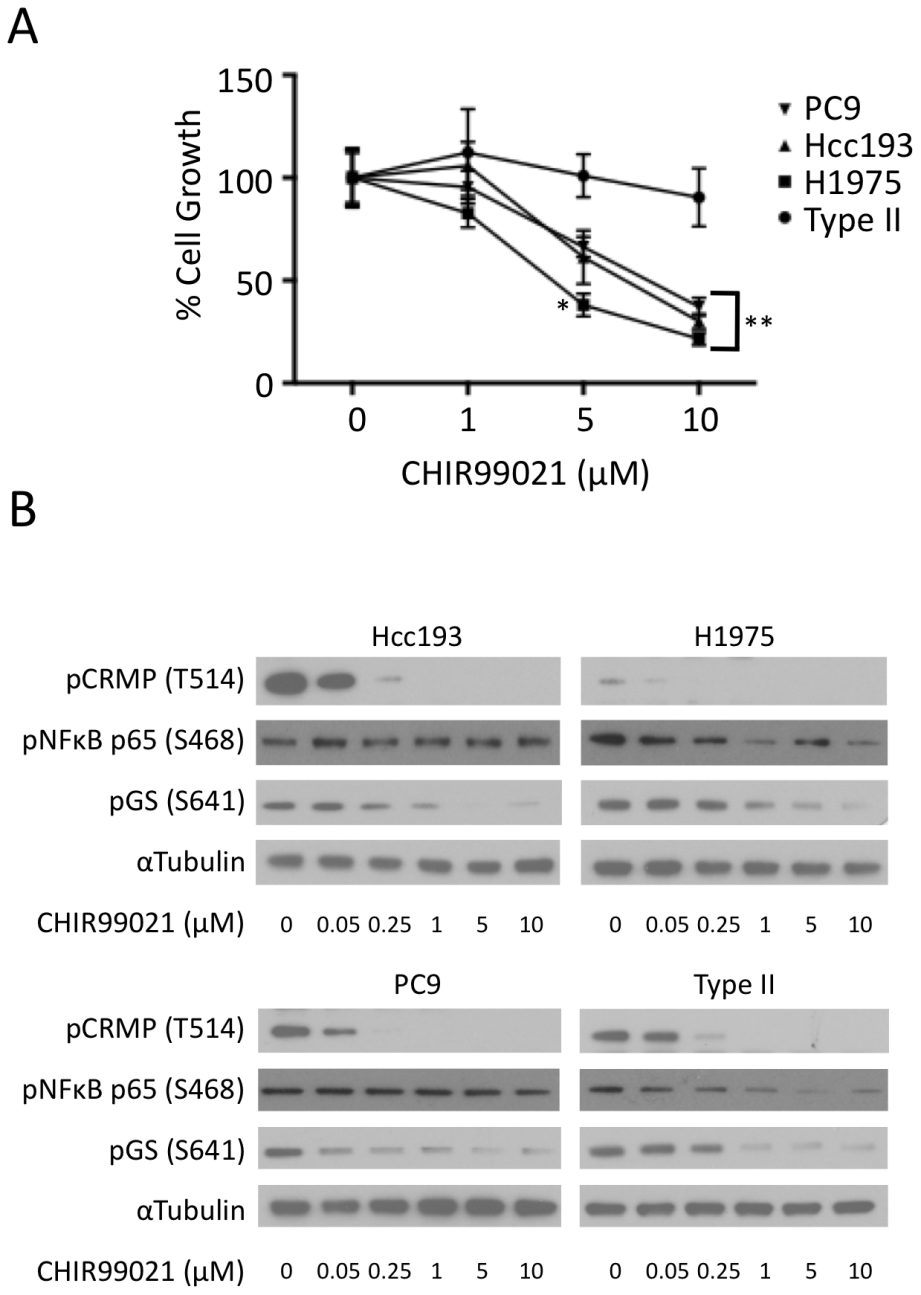
Inhibition of GSK3 suppressors cell proliferation in NSCLC cells. A: NSCLC cells (PC9, H1295 and Hcc193) and type II pneumocytes were cultured for 120 h in the presence or absence of GSK3 inhibitor, CHIR99021 (0, 1, 5 and 10 µM). After 120 h the extent of cell metabolism was assessed using alamar blue. Fluorescence was analysed using a Fusion plate reader and 535 nm excitation and 590 nm emission filters. Each data point represents the average fluorescence over three replicate experiments (mean ± SEM; * indicates p<0.05, ** p<0.001 relative to the growth of type II pneumocytes). B: NSCLC cells (PC9, H1975 and Hcc193) and type II pneumocytes were cultured in the absence and presence of CHIR99021 (0.05, 0.25, 1, 5 and 10 µM). Protein was extracted and lysates were separated on SDS-PAGE gels. Phosphorylation of GS (S641), NFκB p65 (S468) and CRMP (T514) was determined by western blotting. An anti-α-tubulin antibody was used as a control for protein loading.

Cells were cultured in the presence or absence of the GSK3 inhibitor for 120 h. CHIR99021 decreased the proliferation of three NSCLC cell lines in a dose dependent manner. The proliferation of type II pneumocytes was not inhibited (Fig. 6A). In contrast to the other NSCLC cell lines, A549 cell proliferation was not affected by the inhibitor (S4 Figure).

To relate the effect of CHIR99021 on cell proliferation to the inhibition of the kinase itself, the phosphorylation of GSK3 substrates CRMP, NFκB and GS was assessed following cell treatment with the GSK3 inhibitor. In all cell lines (including type II pneumocytes) the phosphorylation of CRMP on T514 and GS on S641 was reduced in a dose-dependent manner by CHIR99021 (Fig. 6B). By contrast the phosphorylation of NFκB on S468 was largely unaffected by the inhibitor in the NSCLC cell lines but was reduced in type II pneumocytes (Fig. 6B) and in A549 cells (S4 Figure).

## Discussion

This study provides the first evidence that GSK3 protein kinase activity is elevated in early stage NSCLC and that this may contribute to the cancer cell phenotype. The expression and protein kinase activity of GSK3 was elevated in 41% of NSCLC tumours in comparison to patient-matched normal tissue derived from the surgical resection margin. In addition, GSK3 was found to positively regulate cell proliferation in three NSCLC cell lines, but not in non-neoplastic type II pneumocytes.

The expression level of GSK3 has been studied in various cancers. For example a reduction has been reported in human squamous and basal cell skin carcinoma [34], whilst in human colorectal and gastric cancer tissue an increase in expression relative to non-neoplastic tissue has been reported [22], [35]. In addition to analysis of GSK3 expression, kinase activity in cancer has been reported on the basis of the phosphorylation status of the kinase. In oral, laryngeal, esophageal and salivary gland cancer [14], breast cancer [36] phosphorylation on the inhibitory serine (GSK3β S9) was reported to be elevated and the activity of GSK3 was reasoned to be reduced as a result.

In NSCLC GSK3β has been recently reported to be over-expressed in tumour tissue compared to normal tissue isolated from the resection margin and this was found to correlate with poor patient prognosis [25]. In a separate study phosphorylation of GSK3α/β on the inhibitory site, S21/9, was also found to be increased in NSCLC tumour tissue compared to normal, and this too correlated with poor patient prognosis [16]. However, in neither study protein kinase activity is measured.

We therefore sought to clarify this discrepancy and our study is the first to determine GSK3 protein kinase activity *in situ* in NSCLC. The increase in GSK3β expression we observed in Group 1 tumours (Fig. 1) was frequently accompanied by an equivalent increased phosphorylation of the inhibitory S21/9 sites (Fig. 4 and S1 Figure). This is consistent with the observations of [25] and [16], respectively. In addition to this we demonstrated that GSK3β over-expression was often accompanied by GSK3α overexpression (Fig. 2). However, when the level of GSK3 phosphorylation was normalized to the level of GSK3 expression (S3 Figure), Group 1 tumours no longer displayed significantly elevated GSK3 serine phosphorylation (Fig. 5). Furthermore, and most importantly, when we measured GSK3 protein activity *in situ* through examination of the phosphorylation of three distinct proteins on well established GSK3 phosphorylation sites (glycogen synthase (S641/645), CRMP (T514) and NFκB p65-RelA (S468); Figs 3–5), it was increased in the tumours compared to patient matched control tissue. Our data therefore show that activity of the kinase cannot be inferred from serine phosphorylation state alone and that phosphorylation of GSK3 substrates should be measured to properly assess GSK3 activity. Consistent with this, the degree of GSK3α/β inactivation by serine phosphorylation on S21/9 is only approximately 50% [37]. Furthermore, Lim et al have shown that the phosphorylation status of GSK3 substrates (including CRMP) is inconsistent with the level of phosphorylation of GSK3β at serine 9 [38]. Our data showing elevated GSK3 protein kinase activity in NSCLC is, therefore, an important advance in understanding the role of GSK3 in NSCLC.

It is of interest to note that NFκB, one of the GSK3 substrates used here as an indicator of GSK3 activity, is a transcription factor involved in the regulation of cell proliferation, differentiation and apoptosis, is deregulated in many tumours [39] and can be activated through phosphorylation of the p65-RelA subunit by GSK3 [40]. GSK3 inhibition has been shown to be accompanied by a reduction in NFκB activity in osteosarcoma [23], renal cell carcinoma [24], glioma [21], pancreatic [17], [41], [42] and chronic lymphocytic leukaemia [35] cell lines. However, in our study we found that phosphorylation of NFκB was insensitive to GSK3 inhibition in NSCLC cell lines sensitive to CHIR99021, suggesting GSK3 activity on NFκB is unlikely to be involved in the affect of the kinase on NSCLC cell proliferation/viability. In contrast, phosphorylation of GS and CRMP-2 was decreased upon GSK3 inhibition; this suggests they could be involved in mediating the proliferation/viability of NSCLC.

Having demonstrated an increase in GSK3 activity in human tumour tissue we used NSCLC cell lines to investigate whether GSK3 activity was able to support cellular proliferation. The proliferation of three NSCLC cell lines (Hcc193, H1975 and PC9) was sensitive to GSK3 inhibition in a dose dependent manner using a specific inhibitor of GSK3 (Fig. 6). The proliferation of type II pneumocytes was insensitive to GSK3 inhibition, despite exhibiting inhibitor-sensitive GSK3 activity similar to that found in Hcc193, H1975 and PC9 cells (Fig. 6). This suggests that these NSCLC cells have become dependent on the GSK3 pathway for proliferation during tumourigenesis. Consistent with the role of GSK3 in supporting a tumour phenotype, its inhibition has been shown to promote apoptosis in NSCLC cell lines [43], [44]. By contrast we found that proliferation of another NSCLC cell line, A549 was insensitive to GSK3 inhibition. This is inconsistent with the observation that siRNA-mediated knockdown of GSK3 A549, led to reduced cell proliferation [25], however others have not observed a growth inhibitory affect of CHIR99021 on this cell line [44]. That not all NSCLC cell lines are sensitive to GSK3 inhibition is unsurprising given our human tissue data, as not all patients have upregulated GSK3 activity in their tumour tissue.

The difference we have noted in sensitivity of type II pneumocytes and NSCLC cell lines to GSK3 inhibition allows for the possibility for a therapeutic window in which the proliferation of tumourigenic cells can be inhibited but that of non-neoplastic cells is relatively unaffected. Consistent with this, the growth of HEK293 [22], [35], cultured human mammary epithelial cells (HMEC) and embryonic lung fibroblasts [17] are also insensitive to pharmacological inhibition of GSK3. Furthermore, Shakoori et al have reported that a GSK3 inhibitor was able to block the growth of human colon cancer xenografts in mice without adverse side effects [45].

Despite this, treating patients with inhibitors of a kinase known to act as a tumour suppressor in some pathways (such as the Wnt pathway) represents a potential concern [11]. This is, however, mitigated by the finding that more than a 75% inhibition of GSK3 is required to affect β-catenin phosphorylation and stability [7]. Additionally, a lack of correlation between GSK3 activity and nuclear β-catenin expression has been shown in colorectal [22], pancreatic [46] and gastric cancer [47], and this may also be true in NSCLC [25]. This suggests that GSK3 has a signalling role independent of Wnt in several tumour types. Furthermore, psychiatric patients receiving long-term lithium carbonate (an inhibitor of GSK3) have a significantly reduced risk of cancer than the general population [48]. Taken together these data suggest that GSK3 inhibitors may have the potential to act as effective targeted therapeutics in cancer while demonstrating an acceptable therapeutic window.

To conclude, we provide the first demonstration of elevated GSK3 protein kinase activity in a large subset of human early stage NSCLC and show that NSCLC cell lines depend on GSK3 activity for proliferation. Thus, levels of GSK3 expression and protein kinase activity may facilitate prediction of an anti-tumour response to pharmacological inhibition of GSK3 in individual patients. Importantly GSK3 activity was assessed in NSCLC tissue using known substrates of the kinase; we found activity could not be assumed on the basis of serine phosphorylation alone. This finding has very important implications for the interpretation of previous studies and of those going forward. Our findings further indicate the potential of treating NSCLC patients with currently available GSK3 inhibitors.

## Supporting Information

S1 Figure **Validation of the Luminex X-MAP Technology assay.** A: Lysate from human lung tumour tissue from one patient was analysed using Luminex (xMAP) technology to determine the level of GSK3β expression. The experiment was performed in tripliacte and each bar represents the average expression (mean ± SEM). Lysate containing increasing amounts of protein were analysed. The result was validated by western blotting. B: A549 cells were cultured in the absence and presence EGF (25 ng/ml) alone and EGF with AKTi (10 µM). Protein was extracted and lysates were either analysed using Luminex (xMAP) technology or separated on SDS-PAGE gels to determine the level of GSK3α/β phosphorylation.
(TIFF)

S2 Figure **Phosphorylation of GSK3α/β on S21/9 in NSCLC tumour tissue in comparison to patient-matched normal lung tissue.** Three samples from patient-matched normal (N1-3) and tumour (T1-3) tissues were analysed using Luminex (xMAP) technology to determine the level of GSK3α/β phosphorylation. A: Quantified data for all 29 patients. Each bar represents the average phosphorylation for normal (N1-3; light grey) or tumour (T1-3; black) tissue for each patient (mean ± SEM). The strength of evidence for a difference in phosphorylation between the normal and tumour samples was determined by a Kruskal-Wallis test, and * indicates p<0.05. B: The percentage change in GSK3 phosphorylation in tumour samples in comparison to patient-matched normal tissue where patients are ranked in order of the extent of the percentage change in expression.
(TIFF)

S3 Figure **Normalisation of GSK3α/β phosphorylation on S21/9 to total GSK3 in NSCLC tumour tissue in comparison to patient-matched normal lung tissue.** GSK3α/β phosphorylation (S2 Figure) was normalised to levels of total GSK3 (Fig. 1). A: Quantified data for all 29 patients. Each bar represents the average normalised phosphorylation for normal (N1-3; light grey) or tumour (T1-3; black) tissue for each patient (mean ± SEM). The strength of evidence for a difference in phosphorylation between the normal and tumour samples was determined by a Kruskal-Wallis test, and * indicates p<0.05. B: The percentage change in normalised GSK3 phosphorylation in tumour samples in comparison to patient-matched normal tissue where patients are ranked in order of the extent of the percentage change in expression.
(TIFF)

S4 Figure **Inhibition of GSK3 does not suppress cell growth in A549 cells.** A: A549 NSCLC cells were grown for 120 h in the presence or absence of GSK3 inhibitor, CHIR99021 (0, 1, 5 and 10 µM). After 120 h the extent of cell metabolism was assessed using alamar blue. Fluorescence was analysed using a Fusion plate reader and 535 nm excitation and 590 nm emission filters. Each data point represents the average fluorescence over three replicate experiments. B: A549 cells were cultured in the absence and presence of CHIR99021 (0.05, 0.25, 1, 5 and 10 µM). Protein was extracted and lysates were separated on SDS-PAGE gels. Phosphorylation of GS (S641), NFκB p65 (S468) and CRMP (T514) was determined by western blotting. An anti-α-tubulin antibody was used as a control for protein loading.
(TIFF)
