# Supplementary figures and images for: Glycogen Synthase Kinase 3 Protein Kinase Activity Is Frequently Elevated in Human Non-Small Cell Lung Carcinoma and Supports Tumour Cell Proliferation
Source: PLoS One. 2014 Dec 8;9(12):e114725. doi: 10.1371/journal.pone.0114725 (PMC4259366; doi:10.1371/journal.pone.0114725)

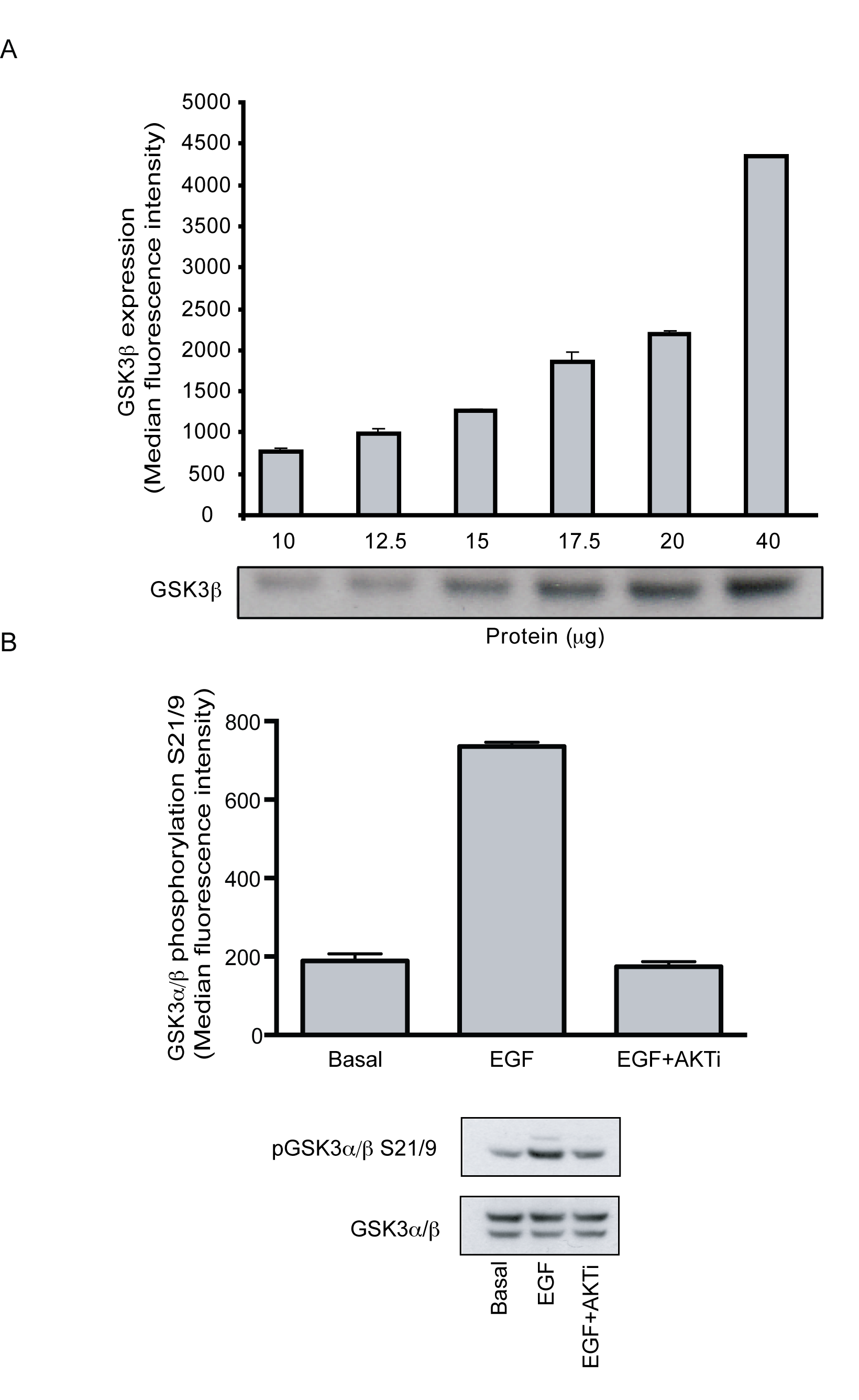

Supplement: S1 Figure — Validation of the Luminex X-MAP Technology assay. A: Lysate from human lung tumour tissue from one patient was analysed using Luminex (xMAP) technology to determine the level of GSK3β expression. The experiment was performed in tripliacte and each bar represents the average expression (mean ± SEM). Lysate containing increasing amounts of protein were analysed. The result was validated by western blotting. B: A549 cells were cultured in the absence and presence EGF (25 ng/ml) alone and EGF with AKTi (10 µM). Protein was extracted and lysates were either analysed using Luminex (xMAP) technology or separated on SDS-PAGE gels to determine the level of GSK3α/β phosphorylation. (TIFF) [file pone.0114725.s001.tiff]

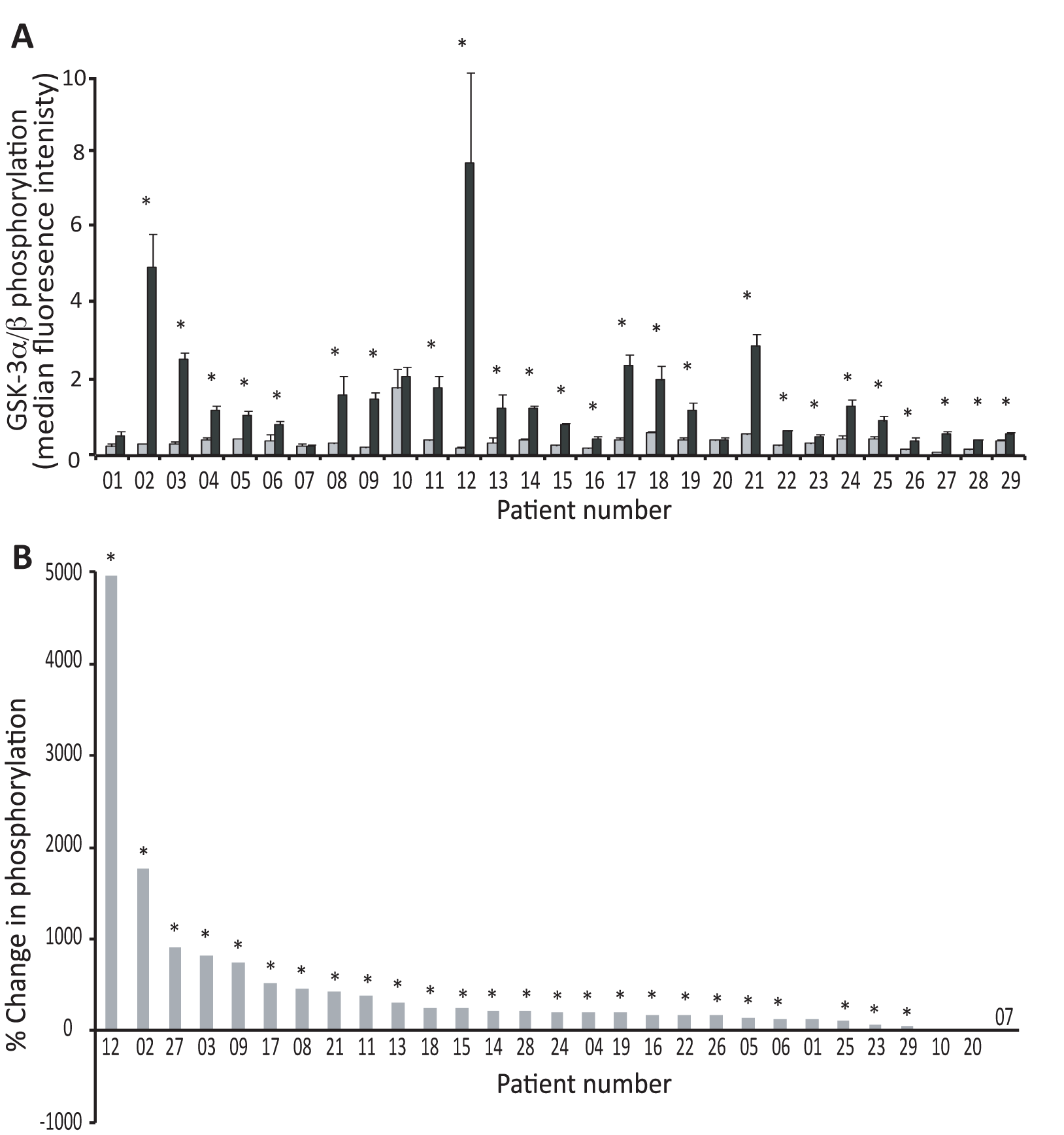

Supplement: S2 Figure — Phosphorylation of GSK3α/β on S21/9 in NSCLC tumour tissue in comparison to patient-matched normal lung tissue. Three samples from patient-matched normal (N1-3) and tumour (T1-3) tissues were analysed using Luminex (xMAP) technology to determine the level of GSK3α/β phosphorylation. A: Quantified data for all 29 patients. Each bar represents the average phosphorylation for normal (N1-3; light grey) or tumour (T1-3; black) tissue for each patient (mean ± SEM). The strength of evidence for a difference in phosphorylation between the normal and tumour samples was determined by a Kruskal-Wallis test, and * indicates p<0.05. B: The percentage change in GSK3 phosphorylation in tumour samples in comparison to patient-matched normal tissue where patients are ranked in order of the extent of the percentage change in expression. (TIFF) [file pone.0114725.s002.tiff]

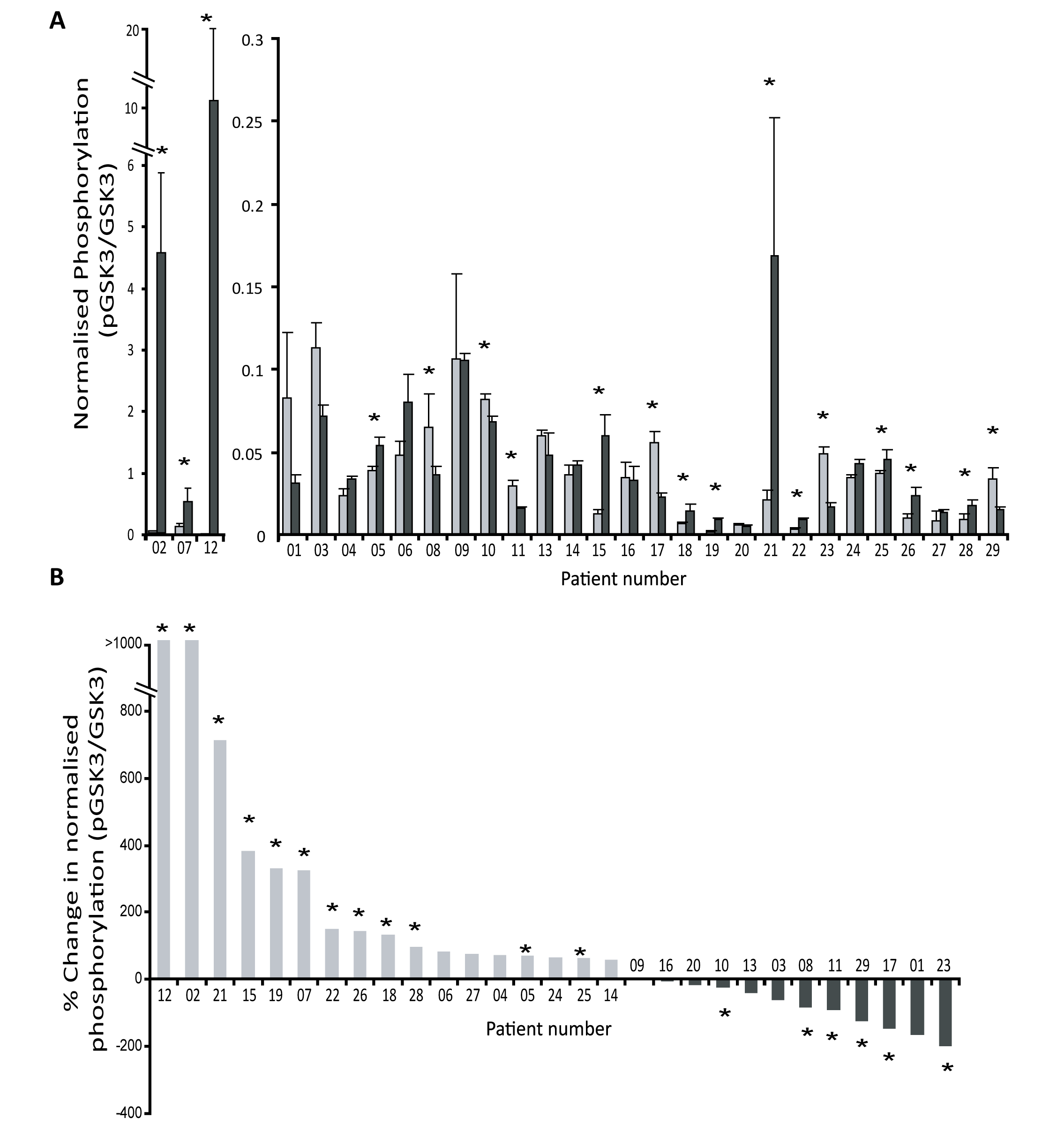

Supplement: S3 Figure — Normalisation of GSK3α/β phosphorylation on S21/9 to total GSK3 in NSCLC tumour tissue in comparison to patient-matched normal lung tissue. GSK3α/β phosphorylation (S2 Figure) was normalised to levels of total GSK3 (Fig. 1). A: Quantified data for all 29 patients. Each bar represents the average normalised phosphorylation for normal (N1-3; light grey) or tumour (T1-3; black) tissue for each patient (mean ± SEM). The strength of evidence for a difference in phosphorylation between the normal and tumour samples was determined by a Kruskal-Wallis test, and * indicates p<0.05. B: The percentage change in normalised GSK3 phosphorylation in tumour samples in comparison to patient-matched normal tissue where patients are ranked in order of the extent of the percentage change in expression. (TIFF) [file pone.0114725.s003.tiff]

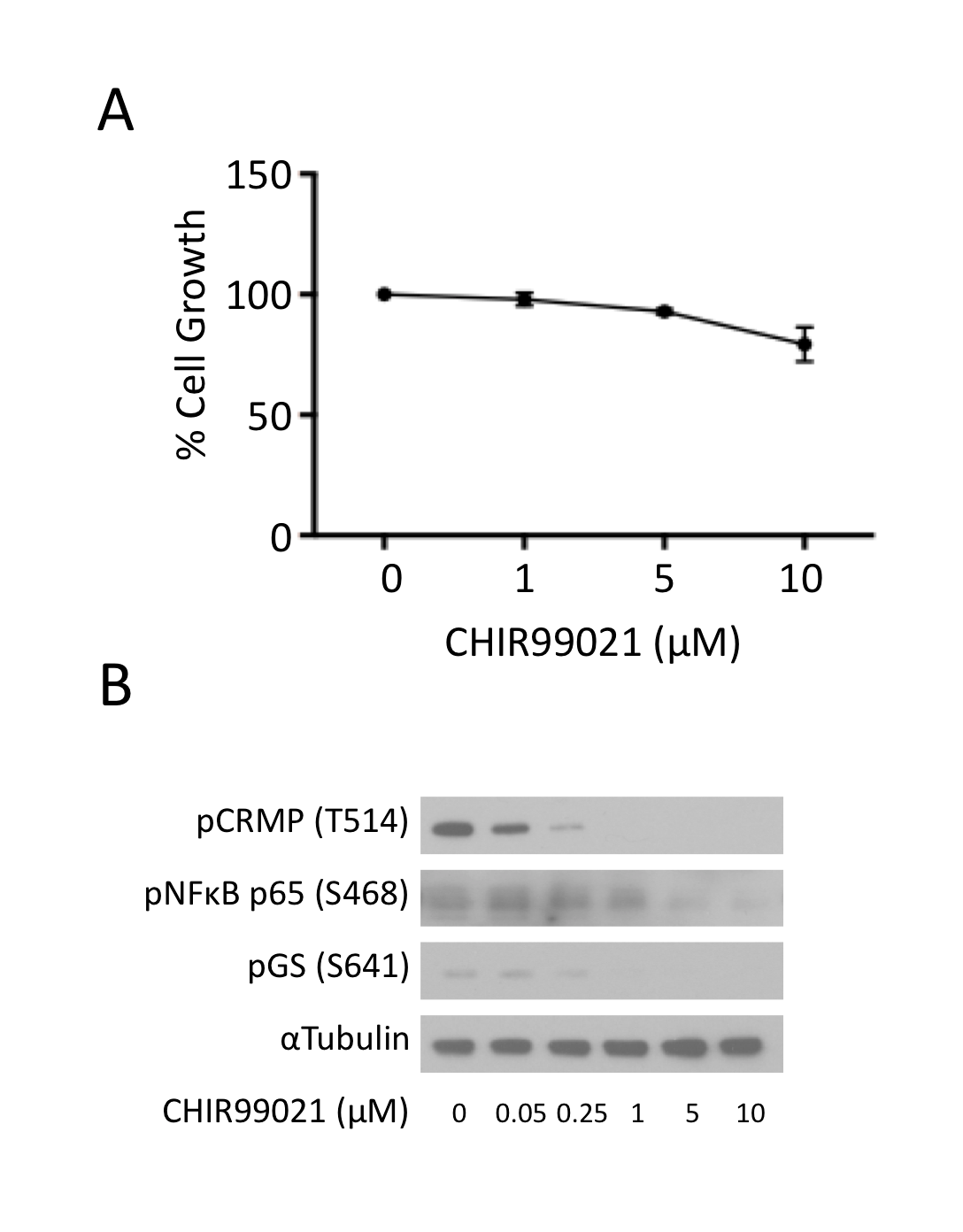

Supplement: S4 Figure — Inhibition of GSK3 does not suppress cell growth in A549 cells. A: A549 NSCLC cells were grown for 120 h in the presence or absence of GSK3 inhibitor, CHIR99021 (0, 1, 5 and 10 µM). After 120 h the extent of cell metabolism was assessed using alamar blue. Fluorescence was analysed using a Fusion plate reader and 535 nm excitation and 590 nm emission filters. Each data point represents the average fluorescence over three replicate experiments. B: A549 cells were cultured in the absence and presence of CHIR99021 (0.05, 0.25, 1, 5 and 10 µM). Protein was extracted and lysates were separated on SDS-PAGE gels. Phosphorylation of GS (S641), NFκB p65 (S468) and CRMP (T514) was determined by western blotting. An anti-α-tubulin antibody was used as a control for protein loading. (TIFF) [file pone.0114725.s004.tiff]
